# Under the dual effect of inflammation and pulmonary fibrosis, CTD-ILD patients possess a greater susceptibility to VTE

**DOI:** 10.1186/s12959-024-00599-3

**Published:** 2024-04-04

**Authors:** Wenli Jiang, Wenhui Jia, Chunling Dong

**Affiliations:** Department of Pulmonary and Critical Care Medicine, Second Hospital, Jilin University, 130041 Changchun, China

**Keywords:** Connective tissue disease, Interstitial lung disease, Venous thromboembolism, Thrombosis

## Abstract

As an autoimmune disease, the persistent systemic inflammatory response associated with connective tissue disease (CTD) is involved in the development of venous thromboembolism (VTE). However, clinical data showed that the risk of VTE in patients differed between subtypes of CTD, suggesting that different subtypes may have independent mechanisms to promote the development of VTE, but the specific mechanism lacks sufficient research at present. The development of pulmonary fibrosis also contributes to the development of VTE, and therefore, patients with CTD-associated interstitial lung disease (CTD-ILD) may be at higher risk of VTE than patients with CTD alone or patients with ILD alone. In addition, the activation of the coagulation cascade response will drive further progression of the patient’s pre-existing pulmonary fibrosis, which will continue to increase the patient’s risk of VTE and adversely affect prognosis. Currently, the treatment for CTD-ILD is mainly immunosuppressive and antirheumatic therapy, such as the use of glucocorticoids and janus kinase-inhibitors (JAKis), but, paradoxically, these drugs are also involved in the formation of patients’ coagulation tendency, making the clinical treatment of CTD-ILD patients with a higher risk of developing VTE challenging. In this article, we review the potential risk factors and related mechanisms for the development of VTE in CTD-ILD patients to provide a reference for clinical treatment and prevention.

## Introduction

Connective tissue disease (CTD), also known as collagen vasculopathy, is an autoimmune disease that causes inflammation and systemic organ damage due to the production of circulating antibodies by autoreactive T and B cells. Subtypes of CTD include systemic sclerosis (SSc), rheumatoid arthritis (RA), polymyositis/dermatomyositis (PM/DM), Sjögren’s syndrome (SS), and systemic lupus erythematosus (SLE). CTD can be caused by a transforming growth factor-β (TGF-β)-dependent pathway, involving the respiratory system and leading to varying degrees of pulmonary fibrosis [1]. This type of interstitial lung disease (ILD) is known as CTD-ILD [2]. CTD-ILD is the second most common subtype of ILD in developing countries, after nodular disease [3].

VTE is a disease in which thrombosis in the veins leads to partial or complete obstruction of the lumen, causing a series of clinical symptoms, including deep venous thrombosis (DVT) and pulmonary thromboembolism (PTE). VTE is the third most common cardiovascular disease worldwide, after myocardial infarction and stroke [4]. Of these patients, 27-56% will develop PE and 20-50% will develop post-thrombotic syndrome [5]. As a common clinical chronic disease, VTE is considered to be a major contributor to the worldwide disease burden [6]and one of the most important causes of patient mortality [7]. Statistics have shown that the incidence of VTE and the hospitalization rate of patients are increasing year by year in China [8, 9].

Patients with CTD-ILD often have a poor prognosis, especially compared with patients with ILD alone [10], and VTE, as a common cardiovascular disease, can also affect the quality of survival and prognosis of patients with CTD-ILD. A prospective clinical study found that the incidence of VTE in patients with CTD-ILD was 20% [11]. Although the subtype of ILD is an independent risk factor affecting the development of VTE in patients with ILD [12], existing clinical studies on the occurrence of VTE in patients with ILD have mainly focused on IPF, and there is a lack of large-scale multicenter clinical statistical studies on the clinical characteristics and risk factors for the occurrence of VTE in patients with CTD-ILD, and exploratory studies at home and abroad on the mechanism of thrombosis in this type of patients are equally inadequate.

In this review, we provide a systematic description of the potential risk factors and mechanisms of VTE in CTD-ILD, to improve clinicians’ understanding of the occurrence of VTE in patients with CTD-ILD, and to provide a reference for the prevention of high-risk patients, the selection of therapeutic regimens, and the continuation of subsequent relevant research.

## VTE associated with the systemic inflammatory response

### Immunothrombosis associated with persistent inflammatory activity

#### Inflammatory activity leading to hypercoagulability

**Fig. 1:**
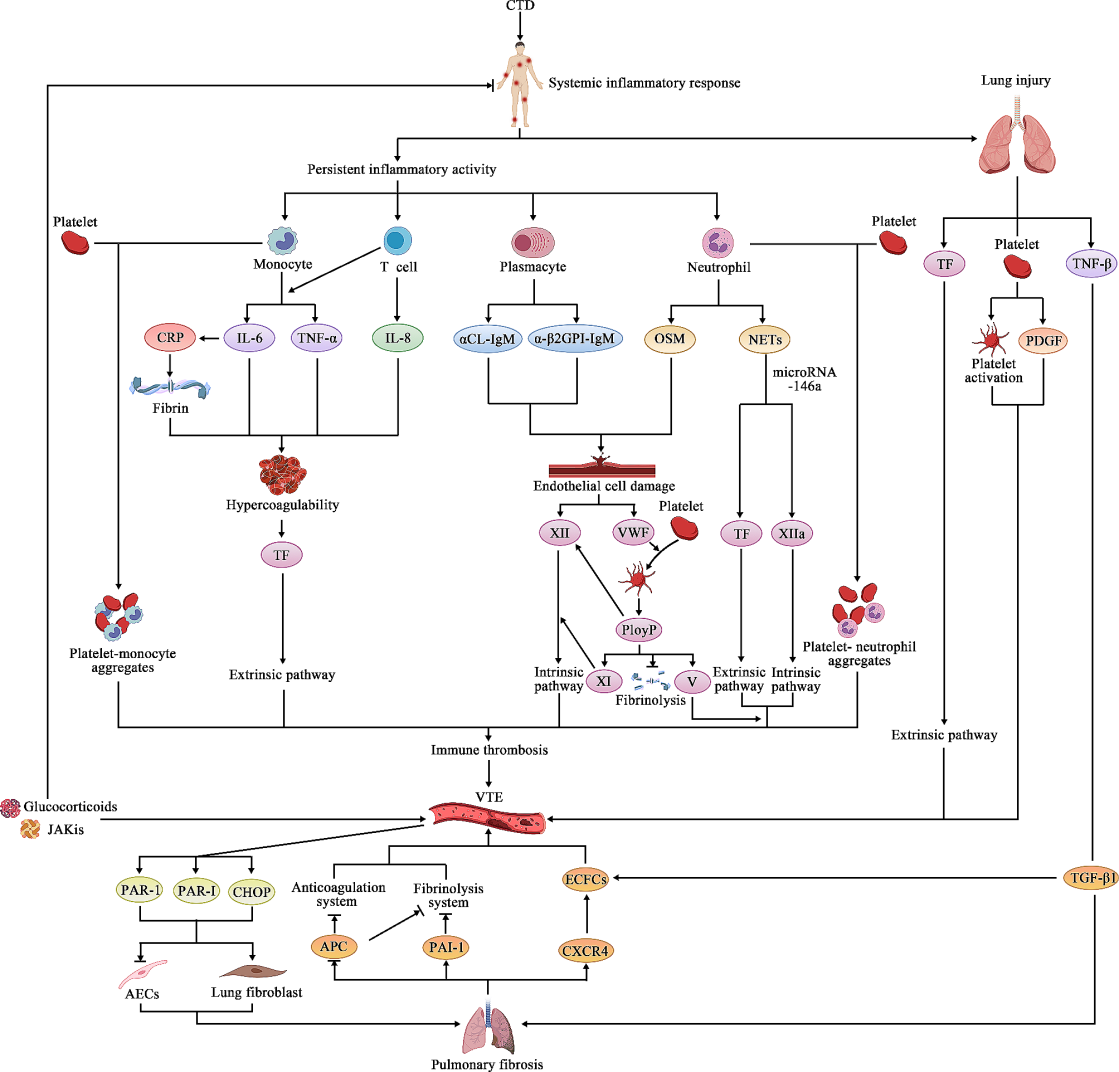
Multiple cytokines are modulated to affect the coagulation system, anticoagulation system, and fibrinolytic system through different pathways and lead to the occurrence of VTE, while at the same time, the activation of the coagulation cascade will exacerbate the original pulmonary fibrosis changes in CTD-ILD patients

As a multi-causal disease, the classical model of VTE etiology was first proposed by Virchow and includes vascular endothelial injury, blood stasis, and hypercoagulable state [13]. Autoimmune-related factors are now considered to play a key role in the development of VTE in patients with CTD-ILD and are additional risk factors for the development of VTE in patients admitted to the hospital [14]. Autoimmune systemic diseases lead to a significant increase in the risk of VTE in the population [15], the cause of which is closely related to persistent inflammatory activity in the body [16]. Currently, some clinical observations have found that the risk of VTE in patients with autoimmune diseases with combined CTD is higher than that in patients without combined CTD [17]. The increased risk of VTE development due to inflammatory activity may be achieved by up-regulating procoagulants, down-regulating anticoagulants, and inhibiting fibrinolysis [18]. In addition, elevated C-reactive protein (CRP), IL-1, IL-6, IL-8, and TNF-α during inflammatory activity have likewise been found to be associated with the development of a hypercoagulable state in vivo [19], when alterations in coagulation may be achieved by inducing the expression of tissue factor (TF), in which IL-6 plays a key role [20]. As a non-specific inflammatory marker produced by the liver, CRP is mainly involved in the increased propensity for thrombosis during short-term acute inflammatory episodes [21], which may be associated with increased fibrin levels in vivo. Although elevated CRP can be used to predict the occurrence of VTE events, it is still controversial whether CRP is directly involved in thrombosis during the active phase of inflammation. Autoimmune patients have a high risk of thrombosis at the beginning of the disease diagnosis, and although this risk decreases with the course of the disease, the hypercoagulable state of the body remains persistent for up to 10 years [22], and the occurrence of inflammatory activity during the course of the disease plays an important role (Fig. 1). In addition to inflammatory markers, D-dimer levels have also been found to be elevated in patients with CTD [23, 24], and this serologic alteration may be closely related to the inflammatory activity of the patient’s rheumatic disease. Inflammatory activity triggers the formation of a hypercoagulable state with concomitant changes in D-dimer [25]. Several investigators have confirmed the correlation between D-dimer levels and rheumatoid arthritis activity indices through retrospective data mining [26]. In addition to its predictive value for the diagnosis of VTE in CTD patients [27], it has been shown that D-dimer level is also a valid serologic index for assessing disease activity in some CTD patients [28, 29]. D-dimer can be used as one of the effective monitoring indicators during the suppression of hypercoagulability due to immune activity with glucocorticoids. Therefore, the occurrence of VTE events may provide some degree of reference for clinical judgment of whether a patient’s rheumatic disease is in an active stage, and similarly, the degree of risk of VTE occurrence should be assessed in patients in an active inflammatory stage, and D-dimer may be an effective serologic evaluation indicator for simultaneously assessing inflammatory activity and the risk of VTE in patients with CTD-ILD.

#### Inflammatory activity leading to endothelial cell damage

Endothelial cells cover the surface of all blood vessels and play an important role in preventing thrombosis and accelerating thrombolysis. Similar to the pathogenesis of thrombotic microangiopathy in patients with autoimmune diseases [30], acute and chronic systemic inflammation can affect the normal function of endothelial cells in arterial and venous blood vessels, resulting in impairment of the physiological anticoagulant, antiplatelet aggregation, and vasodilatory functions of the vascular endothelium [31], which promotes the formation of a hypercoagulable state in patients with CTD, and increases the risk of VTE. A sustained inflammatory response will activate neutrophils in vivo, and activated neutrophils rapidly affect endothelial cell function by secreting the cytokine oncostatin M (OSM) [32]. In addition, the integrity of the body’s vascular endothelium is damaged and a prothrombotic state is formed in response to systemic inflammatory reactions such as elevated IgM class anticardiolipin antibodies (aCL-IgM) and IgM class anti-β2-glycoprotein I antibodies (a-β2GPI-IgM) [33] in SLE patients. After the vascular endothelial integrity is impaired, the activated coagulation factor XII will further upregulate the expression of IL-6, IL-8, and TNF-α to promote the formation of DVT by activating PI3K/AKT signaling [34], which is accompanied by an increase in vWF release. Under the influence of multiple factors such as the weakening of the normal anticoagulant function of vascular endothelial cells, the disruption of the integrity of the vascular endothelium itself, and the formation of hypercoagulability states, the tendency of VTE formation occurs in patients with autoimmune diseases (Fig. 1), so controlling the underlying acute or chronic systemic inflammatory response in the clinic is the key to preventing the occurrence of VTE.

#### Platelets activation and immune thrombosis

Inflammation is the early defense response of the body’s autoimmune system to stimuli and injury, and the occurrence of inflammation can affect coagulation factor levels [35] and lead to a hypercoagulable state of the blood and the formation of immune thrombi, thus preventing the spread of infection [36]. In contrast to other mechanisms of vascular trauma, systemic inflammation will activate the formation of immune thrombi on intact venous vessels when there are abnormalities in the body’s autoimmunity [37]. The process may involve the emergence of activation of endothelial cells, platelets, and leukocytes, the persistent activity of autoinflammation, and the initiation of particle formation, which in turn triggers the activation of the coagulation system by inducing TF. Platelets have been found to be involved in immunothrombosis through the formation of platelet-leukocyte aggregates with circulating monocytes or neutrophils [38], and vWF released after vascular endothelial cell injury mediates platelet activation during this process [39]. In addition, the release of polyphosphate (polyP) during platelet activation can activate the coagulation factor XII which in turn initiates the intrinsic pathway of coagulation and the kinin-releasing enzyme-kinin system [40]. polyP has also been found to be involved in immune thrombosis in patients with autoimmune disorders in vivo by enhancing the activation of coagulation factor V and coagulation factor XI by thrombin and decreasing the degradation of fibronectin [41]. Inflammation-induced activation of neutrophils and formation of neutrophil extracellular traps (NETs) as an innate defense mechanism is also involved in immunothrombosis by inducing TF expression [42] and activation of XIIa-dependent endogenous coagulation pathways [43]. MAZETTO et al. in a study about patients with thrombotic antiphospholipid syndrome found that the release of NETs in patients was accomplished by the involvement of various proteins such as basal arginine deiminase (PADI4), neutrophil elastase (ELANE), myeloperoxidase (MPO), etc. [44]., whereas NETs associate immunization with thrombosis mediated by microRNA-146a (miR-146a) [45]. As an abnormal thrombus forms in the body, an immune thrombus can further trigger the occurrence of VTE in patients with autoimmune diseases (Fig. 1), and targeted therapy against the mechanism of immune thrombus formation may become a new idea for the prevention and treatment of VTE in patients with autoimmune diseases in the future.

### VTE associated with lung injury due to inflammatory activity

In addition to the systemic persistent inflammatory response to altered coagulation status, and immune thrombosis, pulmonary involvement in patients with CTD-ILD in response to systemic inflammation, such as the occurrence of lung injury may also be involved in patients with a potentially elevated risk of VTE. Increased TF concentrations in alveolar lavage fluid during disease progression suggest the presence of lung injury [46], and the pulmonary coagulation cascade will be activated through TF-dependent exogenous coagulation pathways, triggering the emergence of a prothrombotic state. Under the continuous stimulation of chronic inflammation, damaged lung epithelial cells and endothelial cells will also participate in thrombus formation through platelet activation and the release of mediators such as platelet-derived growth factor (PDGF) and TGF-β [47]. In addition, some researchers have found an increase in thrombin concentration in alveolar lavage fluid of ILD patients [48], thrombin is a key enzyme in the coagulation cascade reaction, and when lung tissue injury occurs, the body produces a stable, insoluble cross-linked fibrin clot through the activation of the coagulation cascade reaction, which promotes hemostasis of the blood vessels at the site of the injury to avoid aggravation of the lung injury, but this changes the coagulation state of the lungs at the same time (Fig. 1). The above clinical findings provide evidence that lung injury caused by systemic inflammation is involved in the formation of thrombus in CTD-ILD patients and that effective control of persistent inflammation in CTD-ILD patients can reduce the risk of potential VTE in addition to delaying lung involvement.

### VTE associated with immunosuppressive and antirheumatic therapy

#### VTE associated with immunosuppressive therapy

As one of the autoimmune diseases, the treatment of CTD-ILD patients relies on the use of glucocorticoids and various immunosuppressive agents, which can prevent the further occurrence of irreversible lung damage through anti-inflammatory and immunomodulatory effects [49], thus improving the prognosis of the patients and the quality of their survival. The use of glucocorticoids during immunosuppressive therapy will increase the risk of VTE [50], and other immunosuppressive agents such as Mycophenolate mofetil (MMF) [51] and cyclophosphamide [52] are currently unable to clarify whether or not they have an effect on hypercoagulability in CTD-ILD patients in vivo. Receipt of intravenous immunoglobulin (IVIG) therapy has likewise not been observed to be significantly associated with increased occurrence of VTE in DM patients [53]. Glucocorticoids contribute to the development of a hypercoagulable state in patients by inducing an increase in the levels of procoagulant factors such as factor VIII and vWF, as well as a decrease in fibrinolytic capacity [54]. The degree of VTE risk in CTD-ILD patients treated with glucocorticoids is closely related to the dose and duration of hormone application, and prolonged, higher-dose use of glucocorticoids will lead to a significant increase in the risk of VTE in patients [55, 56]. It is worth mentioning that it is not clear whether the type of glucocorticoid and the way it is used affects the alteration of thrombosis tendency in patients, which can be explored in future clinical studies. In the real world to control the progression of systemic autoimmune disease and respiratory involvement in patients with CTD-ILD, glucocorticoids are recommended as empirical first-line agents, especially during hospitalization for acute exacerbations, and high-dose glucocorticoid shock therapy is often used to improve the symptoms of respiratory distress and progression of the patient’s disease [57]. As the first-line drug for immunosuppressive therapy in CTD-ILD patients, glucocorticoids cannot be ignored for their anti-inflammatory and disease-control effects, along with the side effects of coagulation abnormalities [58]. When glucocorticoids are not selected, the systemic persistent inflammatory activity inherent in CTD disease will likewise increase the risk of VTE in patients after admission. Therefore, during the treatment of these patients, especially during the acute progression of the disease, whether to apply glucocorticoids and the choice of the actual glucocorticoids treatment regimen should be based on the results of clinical VTE risk assessment, and at the same time, in the process of applying glucocorticoids in the patients, the relevant predictive indexes of the development of VTE should be detected, to be vigilant for the occurrence of high-risk VTE events.

#### VTE associated with disease-modifying antirheumatic therapy

Janus kinase-inhibitors (JAKis) as targeted synthetic disease-modifying antirheumatic drugs (tsDMARAs) for disease palliation that have emerged in recent years, are recommended for disease control in patients with RA, DM, and other types of CTD [59, 60]. A current clinical study found that the use of JAKis can result in an increased risk of VTE in patients compared to the general population [61], especially for those with baseline VTE risk factors [60]. Compared to biological-modifying antirheumatic drugs (bDMARDs), JAKis will increase the risk of VTE by 50-100% when used for the treatment of RA [61], especially for patients applying higher doses [62]. This conclusion differs somewhat from the results of an earlier study based on the U.S. Food and Drug Administration, which did not find a particularly strong association between JAKis and an increased incidence of VTE, but the use of JAKis may still pose a potential risk of pulmonary thrombosis and portal vein thrombosis for patients [63]. Although it is now more widely recognized that altered VTE risk affects the safety of JAKis during treatment, studies of JAKi-induced VTE remain at the clinical observational stage, with a lack of larger clinical data. The mechanism by which JAKis use leads to an increased risk of VTE is similarly unclear and may be related to the role of JAK2 receptors in bone marrow cells and platelet production [64], but as JAKis become more widely used in clinical therapy in the future, related studies will be more in-depth, providing guidance for the selection of antirheumatic treatment regimens for patients at high risk of VTE in CTD-ILD. Although He et al. found that the incidence of VTE in RA patients using methotrexate was higher than that in patients using hydroxychloroquine, the causal relationship between these two used DMARAs and the occurrence of VTE could not be clarified [65], [59, 60], so short-term use of common palliative DMARAs may be effective for early control of disease activity and thus reduction of coagulation tendency, provided that reasonable doses are used, and changes in the risk of VTE induced by long-term use are lacking in Relevant clinical trial studies are lacking. It has been shown that short-term use of biologics can reduce D-dimer levels in patients, which may be achieved by controlling the course of their rheumatic disease as well as their disease activity [66], but the effect of this class of drugs on the long-term coagulation status of patients is currently unclear. For other common DMARAs used for disease palliation, no clinical studies have yet found a significant effect of the drug itself on the increased risk of VTE in patients [67], so short-term use of common palliative DMARAs may be effective for early control of disease activity and thus reduction of coagulation tendency, provided that reasonable doses are used, whereas changes in the risk of VTE induced by long-term use currently clinical experimental studies are lacking. It is worth noting that some clinical observational studies have found that changes in antirheumatic drugs during long-term treatment of patients will significantly increase the risk of VTE, and the risk is closely related to the number of changes in antirheumatic drugs during the treatment period [68], but the reasons and mechanisms behind this lack of more in-depth exploration. In the future, large-scale targeted observational studies can be conducted to further investigate the relationship between the use of DMARAs and the risk of VTE, to guide the selection and determination of antirheumatic treatment regimens for CTD-ILD patients with a high risk of VTE.

## VTE associated with CTD-ILD

### VTE associated with pulmonary fibrosis

#### Altered coagulation status due to pulmonary fibrosis

Pulmonary fibrosis is a typical clinical lung change in patients with CTD-ILD. Pulmonary fibrotic changes are also closely related to the development of VTE, and there may be an overlap of pathways between the two [69]. Evrard SM et al. found that TGF-β1 enhances the viability and migration of endothelial colony-forming cells (ECFCs) in conjunction with pro-fibrosis, which promotes vascularization [70]. As circulating mesenchymal-like cells, fibroblasts mediate the onset of pulmonary fibrosis [71] while also promoting the expression and differentiation of ECFCs through CXCR4 [72]. ECFCs are the most pro-angiogenic progenitor cell type [73] and have been found to act as markers of thrombosis [74] and have been associated with the development of hypercoagulable states in patients with ILD [75, 76]. The decrease in activated protein C (APC) in alveolar lavage fluid and the increase in plasma levels of fibrinogen activator inhibitor-1 (PAI-1) again suggest that fibrotic changes in lung tissue are accompanied by changes in the original anticoagulant balance [77]. Fibrotic changes in the lungs bring about the development of hypercoagulable states and an increased risk of VTE in patients with CTD-ILD (Fig. 1).

#### Coagulation cascade reactions exacerbate pulmonary fibrotic changes

At the same time, activation of the coagulation cascade has been found to exacerbate preexisting pulmonary fibrosis in CTD-ILD patients. Activation of the coagulation cascade will exacerbate pulmonary fibrotic changes in patients by driving the activation of protease-activated receptors (PAR) such as PAR-1 [78]. Atanelishvili et al. found strong expression of CCAAT/Enhancer Binding Protein homologous protein (CHOP) in alveolar epithelial cells (AECs) of patients with SSc-ILD by isolating fibrotic lung tissues from patients with SSc-ILD. This finding demonstrates that thrombin, in addition to PAR-1, induces apoptosis in AECs and promotes lung fibroblast survival by regulating the expression of endoplasmic reticulum stress-specific apoptotic protein CHOP, which in turn drives the continued progression of lung fibrosis in CTD-ILD patients [79]. Bruzova et al. found that high levels of PAR-2 were present in the alveolar lavage fluid of CTD-ILD patients by fiberoptic bronchoscopy under local anesthesia and bronchoalveolar lavage [80]. PAR-2 plays a key role in the proliferation of human lung fibroblasts induced by coagulation factor VIIa (FVIIa) [81], therefore, PAR-2 may similarly induce the progression of the degree of pulmonary fibrosis in CTD-ILD after activation of the coagulation cascade. There is a mutually reinforcing effect between fibrotic changes in the lungs of CTD-ILD and the elevated risk of VTE. Pulmonary fibrosis induces VTE at the same time as the coagulation cascade response will exacerbate the pulmonary fibrotic changes through the action of PAR-1 and PAR-2 (Fig. 1). The discovery of the above mechanisms may become a new target for delaying the progression of pulmonary fibrosis and decreasing the emergence of hypercoagulable states in CTD-ILD patients. A single-center, prospective study found that the direct thrombin inhibitor dabigatran reduced thrombin activity in alveolar lavage fluid of SSc-ILD patients and exerted an antifibrotic effect through inhibition of thrombin itself. Although dabigatran was clinically observed to have a good safety and tolerability profile [82], the clinical effect of dabigatran in SSc-ILD patients to slow down the alteration of pulmonary fibrosis has not yet been determined, as well as the clinical effect of dabigatran for other CTD-ILD patients. not determined, as well as the safety and efficacy of dabigatran in other CTD-ILD patients is unclear, and future studies are needed to explore this.

#### VTE associated with severe pulmonary fibrosis

DLCO levels are often used to measure the severity of pulmonary fibrosis in CTD-ILD patients in clinical practice, and ECFCs are now found to be the only endothelial progenitor cell subtype that has a negative correlation with patients’ DLCO levels [83], and, clinically, patients with CTD-ILD tend to be accompanied by lower DLCO levels. Therefore, it is likely that the risk of VTE development in CTD-ILD patients will increase with their increasing lung fibrosis and decreasing DLCO levels [84]. Lower lung function is also thought to be associated with the development of VTE [85]. Severe pulmonary fibrosis and decline in lung function can trigger hypoxia in patients, and hypoxia-inducible factor (HIF) will activate the hypoxia signaling pathway, induce platelet activation and regulate the levels of prothrombotic and antithrombotic factors, which can promote thrombosis and even lead to VTE [86], in which the activation of the nucleotide binding domain, leucine-rich-containing family, pyrin domain containing 3 (NLRP3) inflammatory vesicle complex mediated by HIF-1α plays a key role [87]. Hypoxia will also lead to the downregulation of protein S levels [88] and the promotion of thrombin generation [89], which is involved in the activation of the coagulation cascade. In addition, the enhancement of platelet activity [90] as well as platelet calprotectin activity in plasma [91] under hypoxic conditions is likewise associated with the development of coagulation tendencies. In CTD-ILD patients with severe pulmonary fibrosis, daily activities will be limited due to impaired lung function and hypoxia, and a decline in activity will occur, which will further increase the risk of VTE in this group of patients. Therefore, improving pulmonary function and arterial blood gas analysis, improving the patient’s peripheral oxygenation status, and correcting the decline in arterial partial pressure of oxygen is essential to improve the hypercoagulable state in CTD-ILD patients with severe pulmonary fibrosis and to reduce the risk of VTE in these patients.

### Connective tissue disease subtypes and the occurrence of VTE

#### VTE associated with SSc

SSc is a rare type of CTD clinically characterized by vasculopathy, immune dysfunction, and skin and visceral fibrosis [92]. Among the subtypes of CTD, patients with SSc have the highest risk of developing ILD [93], and patients with SSc-ILD have a high incidence of DVT [94]. In addition to pulmonary changes, SSc as a primary disease is equally involved in the risk of VTE. Compared to the general population, patients with SSc have a 10.5-fold increased risk of DVT and a 7-fold increased risk of PTE [95], especially within the first year after SSc diagnosis [96]. This may be related to the persistent chronic inflammation present in SSc patients leading to arterial and microvascular thrombosis [18], a conclusion supported by the elevated levels of D-dimer found by FURTADO et al. in SSc patients [97]. Higher levels of VWF, VWFpp, and lower levels of ADAMTS-13 in the plasma of patients with SSc indicate the hypercoagulable state that characterizes SSc [23]. Enhanced in vitro thrombin generation in patients with SSc similarly contributes to an increased risk of VTE [98]. SSc often has vasculopathy and vascular injury early in the course of the disease, which limits the normal function of the vascular endothelium and leads to the release of thrombin, triggering the coagulation cascade, which further triggers the patient to be in a procoagulant state. In addition to immune and vascular damage, there is also an association between antiphospholipid antibodies (aPL) [99], which some SSc patients have, and altered risk of thrombosis, and the exact mechanism of occurrence needs to be further explored in the future.

### VTE associated with RA

A large study based on the general population showed that patients with RA had an increased risk of VTE compared with those without comorbid RA, and the risk of VTE was highest during the first year of diagnosis [100], and the risk of VTE in patients would gradually increase during the 10 years after the definitive diagnosis of RA [101]. Mori et al. found that D-dimer was significantly elevated in RA patients with DVT after a long-term follow-up and that clinical disease activity was the main cause of elevated D-dimer in RA patients [102], and a Swedish cohort study also found that the risk of VTE in RA patients was closely related to the degree of rheumatic disease activity [16]. The persistence of clinical disease activity will involve the respiratory system in the development of pulmonary fibrosis, which makes RA-ILD patients similarly at altered risk of VTE.ILD is the most common extra-articular change in RA, and one of the causes of the high mortality rate in RA patients [103]. Therefore, in addition to focusing on their disease activity, attention should be paid to the development of hypercoagulability in patients with RA with rapid pulmonary progression in the clinic, especially in RA-ILD patients with pre-existing ILD changes. In addition to the association with the development of cardiovascular diseases such as myocardial infarction and stroke, et al. found that there is an association between anti-citrullinated peptide 2 (CCP2) antibody positivity and the risk of VTE in patients with RA, and this risk increases with the increase in IgG anti-CCP2 levels [104]. In addition, possible reasons for the increased risk of thrombosis in patients with RA-ILD include limited joint mobility and decreased exercise capacity that affects normal blood flow, undergoing surgery due to the progression of the disease, endothelial damage to blood vessels due to an active inflammatory response, and increased blood coagulability [18]. In the absence of prophylactic measures, surgical patients have a higher risk of developing VTE [105], and RA patients have a significantly higher risk of VTE after undergoing spinal surgery than non-RA individuals [106]. Although some patients with RA-ILD undergo surgery due to joint involvement, available studies have found that the risk of VTE is not further increased after arthroplasty in patients with RA-ILD [107], and the incidence of VTE is comparable between patients with RA and those with osteoarthritis after arthroplasty, which may be related to the antiplatelet effect of the patients’ frequent use of nonsteroidal anti-inflammatory drugs [108]. The use of the emerging drug JAKis during the treatment of RA-ILD patients will also lead to a greater susceptibility to VTE events [60]. The choice of pharmacologic and surgical treatment regimens for patients with RA-ILD should be adequately weighed to avoid VTE during treatment as much as possible and to improve the long-term prognosis of clinical patients.

### VTE associated with PM/DM

Patients with PM/DM are mainly characterized by chronic muscle inflammation and decreased muscle strength, and the mortality rate is about four times higher than that of the general population [109], PM/DM is clinically rare in patients with CTD. The results of a prospective study conducted by FATHI et al. showed that the prevalence of ILD was as high as 78% in patients with PM/DM [110], and although there is some discrepancy between this result and other similar studies [111, 112], they all indicate that the lungs are the most frequently involved organ in PM/DM patients and that PM/DM patients with ILD have a poorer prognosis compared with PM/DM patients alone [113]. Several studies have demonstrated an increased risk of VTE in patients with PM/DM [114–116], especially within the first 5 years of disease diagnosis [114, 117], the reason for which may be related to the active systemic inflammatory response and the fact that the disease has not been effectively controlled. In addition, DM is a microvascular disease mediated by the involvement of body fluids, so vascular endothelial damage during the course of the disease in DM patients may also play a role in the development of VTE [114], which may contribute to a higher risk of VTE in DM-ILD compared to PM-ILD, but there is a lack of large-scale clinical data to support this. The presence of ILD is often found in patients with PM/DM when the diagnosis is clinically confirmed, so patients with PM/DM-ILD should be alerted to the occurrence of VTE when they present with dyspnea, especially in patients with a clear diagnosis of DM-ILD, to avoid further impact on their prognosis. Anti-melanoma differentiation-associated gene 5 (MDA5)-positive DM-ILD is a rare subtype of DM-ILD characterized by acute progressive ILD. Pulmonary changes in these patients progress rapidly, and patients’ activity is often significantly limited [118], to enable patients to achieve a long survival after the onset of the disease, triple immunosuppressive therapy including high-dose hormones, tacrolimus, intravenous cyclophosphamide is often chosen for treatment, and the emerging drug JAKis may be chosen for refractory patients to improve survival [119], and the above treatment methods may The above treatments may lead to an increase in the thrombotic tendency of patients based on the original, so prophylactic anticoagulation may be given to these patients after the treatment plan is determined, to avoid the occurrence of VTE and related adverse complications, which may affect the survival and quality of life of patients.

### VTE associated with SS

SS is an autoimmune disease characterized by generalized dryness, usually including dry skin and eyes. SS has a high prevalence of ILD, and ILD involvement can occur at all stages of the disease course, with ILD manifestations often appearing before other manifestations in some patients [120]. Patients with SS have a higher propensity for thrombosis compared to the general population, and SS is an independent risk factor for the development of VTE in patients [121], which persists for at least 5 years after the diagnosis of SS and is highest when the disease is active and the systemic inflammatory response is poorly controlled [122]. In a study stratified based on autoantibody test results, patients who were positive for both anti-Ro/SSA and anti-La/SSB antibodies had a higher relative risk of VTE [123]. In addition to inflammatory activity factors, the increased risk of VTE in some SS patients may be related to their aPL. aPL can be involved in thrombosis by stimulating exogenous coagulation pathways, platelet aggregation, activation of complement, and inhibition of the anticoagulant activity of activated protein C and protein S [124]. The presence of aPL can be detected in more than one-third of patients with primary SS (pSS) [125], in which lupus anticoagulant (LAC) has been suggested to serve as an important marker for the development of VTE in pSS patients [126], in addition, LAC is the most valuable aPL for predicting thrombotic events in patients with CTD [127]. There are fewer studies related to the causes and mechanisms of the increased thrombotic tendency in SS patients, and the effect of SS in SS-ILD patients with enhanced procoagulant response in vivo should not be ignored and should be of interest to a wide range of investigators.

### VTE associated with SLE

SLE is predominantly found in women of childbearing age and can present with multiple organ involvement [128], and patients are clinically less likely to present with ILD. A 10-year study in France showed that ILD was present in approximately 1.2% of SLE patients, and ILD was a major risk factor for death in SLE patients [129]. Despite the relatively small proportion of SLE-ILD patients in the total SLE population, the higher risk of VTE and mortality that patients have is still a cause for concern. Compared with the general population, patients with SLE have a 3- to 4-fold increased risk of VTE, and the risk of VTE is highest in the first year after a definitive diagnosis of SLE [130]. When SLE patients are hospitalized and treated for VTE, patient mortality is significantly higher, and for SLE patients hospitalized for non-VTE reasons, the risk of VTE during hospitalization is likewise further increased [131]. The abnormalities of thrombosis in SLE patients may be closely related to their antiphospholipid antibodies (aPL) and genetic polymorphisms [132], in which LAC is involved in the formation of hypercoagulable state in SLE patients [127]. Decreased degradation of NETs due to decreased activity of DNase1 in SLE patients may also lead to an increased tendency to thrombosis [133], therefore, targeted NETs therapy may be a new option for the clinical prevention and treatment of VTE in SLE patients. It has been found that VTE is common at the beginning of the SLE course and is strongly associated with disease activity, especially when patients have vasculitis, nephrotic syndrome, hormonal therapy, or LAC [134]. In addition, SLE patients have a higher prevalence of hypertension, diabetes mellitus, and hyperlipidemia than the general population, which can likewise contribute to an increased risk of VTE in patients [135]. The risk of VTE is further increased after undergoing surgical treatment for complications of SLE [136]. The causes of VTE in patients with SLE are complex, and in addition to autoimmune factors, a variety of factors such as renal disease, the use of therapeutic drugs such as non-steroidal anti-inflammatory drugs (NSAIDs), and surgical procedures are involved, and the interaction of the factors ultimately puts the patient at high risk for the development of VTE.

## Other factors with the occurrence of VTE

The occurrence of VTE in CTD-ILD patients is associated with a variety of factors. The role of genetic factors in thrombotic tendency has been widely confirmed, such as coagulation factor V Leiden mutation, prothrombin G20210A mutation, etc [137]. Among them, the coagulation factor V Leiden mutation is now thought to be possibly associated with patients with CTD-ILD, and patients with coagulation factor V Leiden mutation are often accompanied by severe respiratory distress and decreased lung function [138]. Coagulation factor V Leiden mutation is a common cause of primary thrombosis and is associated with increased resistance of coagulation factor V to activated protein C [139]. Statistically, patients with coagulation factor V Lei mutation will have an 18-fold increased risk of VTE compared to the general population [140]. Therefore, when CTD-ILD patients present with increased dyspnea and VTE, there may be a common driver for both. Although existing studies have found a low incidence of the coagulation factor V Leiden mutation in patients with CTD, and it is not possible to confirm whether there is a direct association between this mutation and the development of VTE in patients with CTD-ILD [141], patients with CTD-ILD who are positive for the coagulation factor V Leiden mutation should be highly vigilant for the development of VTE in the clinic, especially when the patients present with severe dyspnea.

**Fig. 2:**
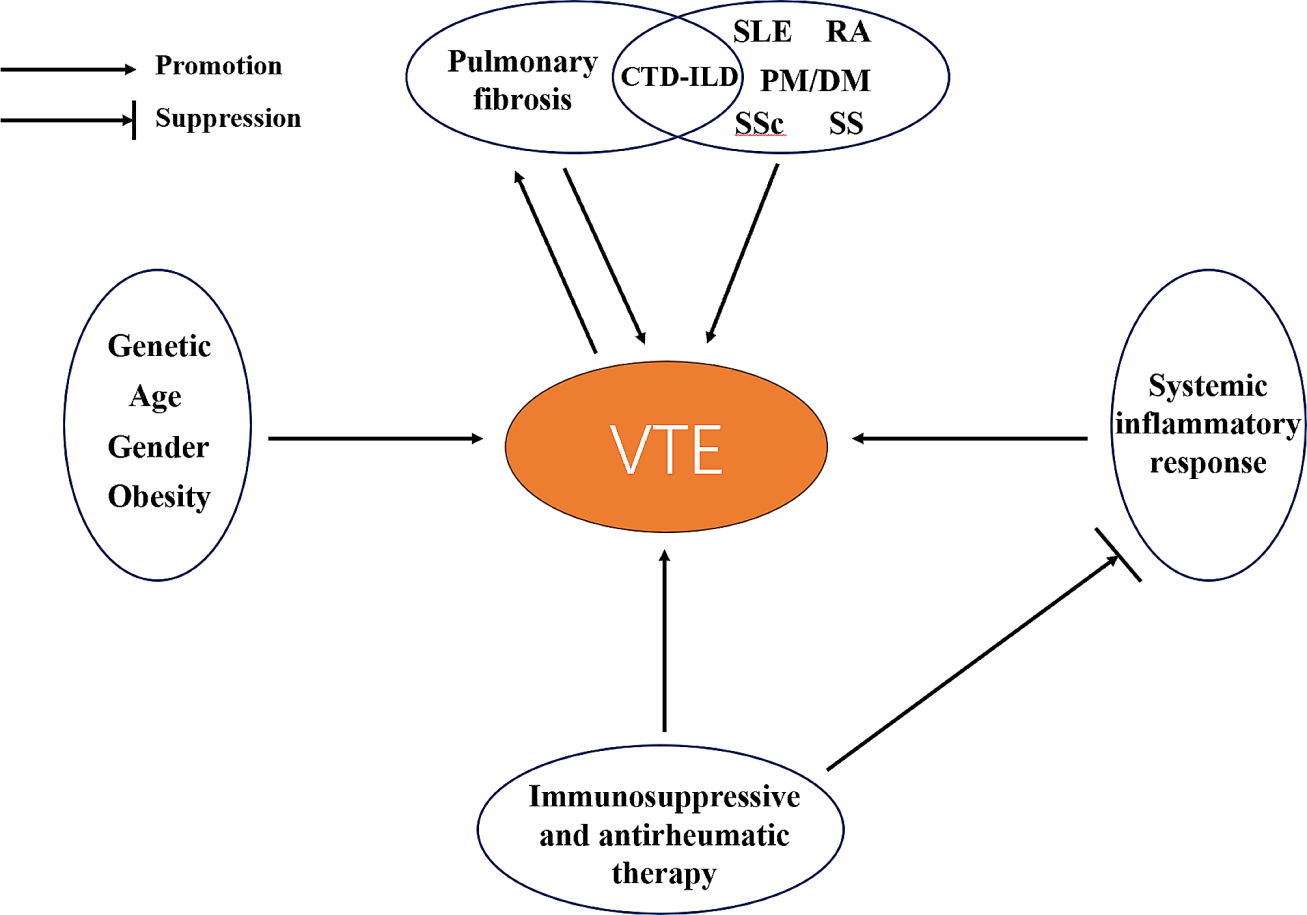
Multiple factors are associated with the occurrence of VTE

As with other cardiovascular diseases, the annual incidence of VTE is closely related to age, and older age at diagnosis is considered to be one of the risk factors for the increased incidence of VTE in patients with CTD-ILD [84], and the incidence of VTE continues to increase with age [142], and the results of a single-center study based on a single-center study showed that the average age of patients with CTD-ILD was 55.6 years old [143], with increasing age, the balance of coagulation factors and coagulation inhibitors in the patient’s body will change [144], and the coagulation pathway is activated [145] making the blood in a hypercoagulable state. Vascular endothelial function and regrowth capacity will also change with age [146]. This ultimately leads to an increased risk of VTE in patients with CTD-ILD, affecting patient prognosis and quality of survival. Although there is no significant difference between the incidence of VTE and gender in the general population [142], the gender difference can affect the risk of VTE recurrence in patients, and studies have shown that men have a higher risk of VTE recurrence [147], Therefore, clinicians should be more concerned about the risk of VTE recurrence that male CTD-ILD patients have after the occurrence of VTE compared to female CTD-ILD patients. In addition, obesity, one of the side effects of hormone use, has likewise been found to be associated with VTE recurrence, and obesity is a time-dependent risk factor for VTE [148]. For CTD-ILD patients, the causes of their increased risk of VTE are multifaceted (Fig. 2) and need to be assessed and judged comprehensively based on the real situation of real-world individual patients.

## Conclusion

As an autoimmune disease, systemic acute and chronic inflammatory responses play an important role in the increased thrombotic tendency of patients with CTD-ILD, and some immunosuppressive and antirheumatic therapies have been found to increase the risk of VTE in patients while suppressing inflammation and slowing down the disease. In addition, risk factors specific to each subtype of CTD are also involved in the generation of hypercoagulability in vivo, and differences in these risk factors may influence the incidence of VTE in each subtype of CTD-ILD, but the clinical statistical analysis is lacking for this speculation. Therefore, patients with CTD-ILD, especially those in the active inflammatory phase and disease progression, need to be adequately evaluated for their own risk of VTE as well as changes in VTE risk that may be brought about by the therapeutic process, based on the patient’s autoimmune disease condition, when choosing the therapeutic regimen.

CTD-ILD is one of the most common types of ILD, and the respiratory involvement of CTD-ILD, such as the occurrence of lung injury, changes in lung fibrosis, and the decline in lung function, is also involved in the increased risk of VTE. At the same time, the activation of the coagulation cascade will drive the activation of PAR to exacerbate the patient’s pulmonary fibrotic changes. The interaction between hypercoagulable state and altered lung fibrosis may in the future be a new target for clinical CTD-ILD patients to receive VTE risk reduction therapy while delaying lung fibrosis. Although some scholars have found that dabigatran, as a direct thrombin inhibitor, can inhibit fibrotic changes in the lungs of patients by inhibiting thrombin activity, the specific anti-fibrotic effect and safety of dabigatran when used in patients with CTD-ILD lacks the proof of long-term clinical trials at present, and therefore it cannot be used as a therapeutic option for large-scale use in patients with CTD-ILD in the clinic.

Under the combined effect of multiple risk factors (Fig. 2), the tendency of CTD-ILD patients to thrombosis is altered, and some of them eventually develop VTE, which adversely affects the quality of survival and prognosis of these patients; therefore, clinicians need to pay extensive attention to the coagulation status of CTD-ILD patients and take appropriate clinical interventions when necessary. Although there are few studies on the occurrence of VTE in CTD-ILD patients at home and abroad, the subsequent large-scale multicenter clinical data study of CTD-ILD patients and the in-depth study of the risk factors and mechanisms of thrombosis in these patients may provide new ideas and solutions for the clinical prevention and treatment of CTD-ILD combined with VTE in the future.

## Data Availability

No datasets were generated or analysed during the current study.

## Abbreviations

CTD: connective tissue disease
ILD: interstitial lung disease
VTE: venous thromboembolism
DVT: deep venous thrombosis
PTE: pulmonary thromboembolism
JAKi: Janus kinase-inhibitors
SSc: systemic sclerosis
RA: rheumatoid arthritis
PM/DM: polymyositis/dermatomyositis
SS: Sjögren’s syndrome
SLE: systemic lupus erythematosus
TGF-β: transforming growth factor-β
CRP: C-reactive protein
TF: tissue factor
NETs: neutrophil extracellular traps
PAR: protease-activated receptors
AECs: alveolar epithelial cells
